# Intensity matters: Therapist-dependent dose of spinal transcutaneous electrical nerve stimulation

**DOI:** 10.1371/journal.pone.0189734

**Published:** 2017-12-15

**Authors:** Diego Serrano-Muñoz, Julio Gómez-Soriano, Elisabeth Bravo-Esteban, María Vázquez-Fariñas, Julian Taylor, Juan Avendaño-Coy

**Affiliations:** 1 Sensorimotor Function Group, Hospital Nacional de Parapléjicos, Toledo, Spain; 2 Toledo Physiotherapy Research Group (GIFTO), Nursing and Physiotherapy School, Castilla La Mancha University, Toledo, Spain; 3 Hospital Nacional de Parapléjicos, SESCAM, Toledo, Spain; 4 Stoke Mandeville Spinal Research, National Spinal Injuries Centre, Buckinghamshire Healthcare Trust, NHS, Aylesbury, United Kingdom; 5 Harris Manchester College, University of Oxford, Oxford, United Kingdom; University of Ottawa, CANADA

## Abstract

The intensity used during transcutaneous electrical nerve stimulation (TENS) in both, clinical practice and research studies, is often based on subjective commands such as “strong but comfortable sensation”. There is no consensus regarding the effectiveness dose of TENS. The objective was to determine the difference in the effect of spinal TENS on soleus H-reflex modulation when applied by two therapists instructed to apply the stimulation at a “strong but comfortable” intensity. Twenty healthy volunteers divided into two groups: Therapist 1 (n = 10) and Therapist 2 (n = 10). Both therapist applied **s**pinal TENS and sham stimulation at the T10–12 spinal level for 40min in random order to each subject, at an intensity designed to produce a “strong but comfortable” sensation. To avoid habituation, the intensity was adjusted every 2min. Soleus H-reflex was recorded before, during, and 10min after TENS by an observer blinded to the stimulus applied. Despite the instruction to apply TENS at a “strong comfortable” level, a significant difference in current density was identified: Therapist 1 (0.67mA/cm^2^, SD 0.54) applied more than Therapist 2 (0.53mA/cm^2^, SD 0.57; *p<*0.001) at the onset of the intervention. Maximal peak-to-peak H-reflex amplitude was inhibited significantly more 10min following TENS applied by Therapist 1 (-0.15mV, SD 0.16) compared with Therapist 2 (0.04mV, SD 0.16; *p* = 0.03). Furthermore, current density significantly correlated with the inhibitory effect on peak-to-peak Soleus H-reflex amplitude 10 min after stimulation (Rho = -0.38; *p* = 0.04). TENS intensity dosage by the therapist based on the subjective perception of the participants alone is unreliable and requires objective standardization. In addition, higher current density TENS produced greater inhibition of the Soleus H-reflex.

## Introduction

Transcutaneous electric nerve stimulation (TENS) is usually applied in the treatment of pain [1], and for sensorimotor dysfunction such as spasticity [2, 3]. The parameters of TENS (amplitude, frequency, pulse width) are modified depending on the pathology [1]. Stimulation frequency and pulse width are important parameters that determine the quantity of electric current administered [1]. Although it has been evidenced that intensity is an essential parameter in determining the dose and the expected effect [1, 4, 5], this parameter is not usually controlled sufficiently as a key factor in both clinical and research practice.

Parameters of the current that influence the dosing of TENS application, such as frequency, pulse width, or intervention time, are expressed using objective values. However, the intensity is usually reported by subjective parameters, such as the intensity that produces a “strong but comfortable” sensation. [6, 7]. In both clinical practice and TENS research studies, this application of intensity in terms of subjective perception could be influenced by the therapist's interpretation. This may be a limitation when comparing the effectiveness of the intervention, especially taking into account that TENS produces intensity-dependent effects [5, 8–12]. Indeed, a systematic review of the effect of TENS applied at a low or barely perceptible intensity presented strong evidence for inefficacy of experimental pain modulation, compared with a “strong but comfortable” TENS intensity where evidence of effectiveness was identified [1]. Interestingly, after calculating the current intensities from previous studies, we observed that different doses had been applied to attain the same current sensation, described as a “strong tingle but comfortable”. For example, Moran et al. [5] used 1.56mA/cm^2^, whereas Vassal et al. [13] applied 0.25mA/cm^2^, and Claydon et al. [10] used 0.44mA/cm^2^. To date, no study has attempted a direct comparison of the current intensity applied and the subjective sensation of the stimulus nor the influence of different therapists on adherence to subjective instructions to define TENS intensity.

Grading TENS intensity based on subjective sensation is also complicated by other phenomena such as habituation, where the perceived intensity of the stimulus decreases over time, which requires periodic increases in the intensity of the stimulus during the course of the TENS session [12]. Stimulus increase during habituation is usually performed during programmed short intervals or when the subject asks that the stimulus be adjusted [5, 9, 12, 14]. Most studies do not perform stimulus intensity readjustment [2, 15, 16]. Furthermore, periodic increases in stimulus intensity during TENS has been shown to produce a greater functional effect when the stimulus was maintained constant from the beginning of the stimulation session [12].

Soleus H-reflex activity has been used as an objective neurophysiological measure of alpha motoneuron excitability [17]. H-reflex activity has been used to diagnose sensorimotor dysfunction such as spasticity [18] and to evaluate changes in spinal cord sensorimotor excitability following intervention [19–24]. The effect of peripheral nerve TENS has been assessed on H-reflex modulation in subjects with spasticity [15] and healthy volunteers [19–25]. The main effect of TENS on H-reflex activity is characterized by inhibition [15, 19], although in other studies, no effect has been identified[20, 22, 23]. Variability of TENS parameters may account for differences observed among studies, especially in stimulus intensity.

In the present study, we hypothesize that, given the same instruction for TENS intensity for evoked sensation, two independent therapists would interpret the same instruction based on a “strong but comfortable” sensation differently. The main objective of this study was, therefore, to quantify TENS intensity applied by two independent therapists who were instructed to apply the same intensity by evaluating the subjective sensation perceived by the participant. The secondary objective was to determine the effect of TENS stimulation applied at the T10–12 spinal level in healthy subjects on Soleus H-reflex amplitude and to assess the effect due to different therapists with the stimulus applied at the same pulse-width and frequency.

## Methods

### Study design

**Fig 1:**
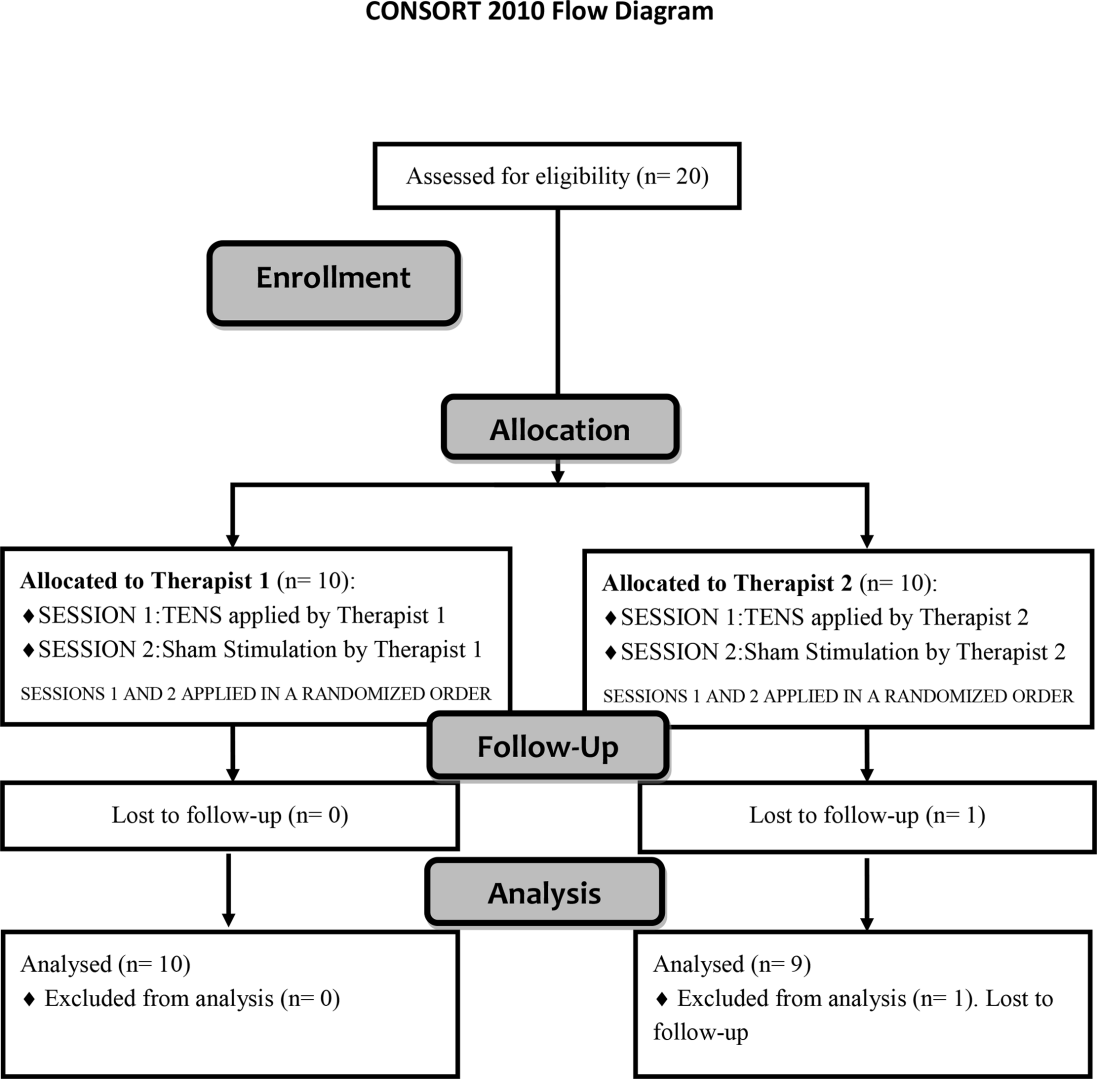
Flow diagram. Recruitment and flow diagram of participants through the trial.

The procedures and design of the trial were approved by the local ethics committee "Complejo Hospitalario de Toledo" (23; 18/02/2011). The trial was registered in ClinicalTrials.gov Protocol Registration System (Number: NCT02718690), it was not registered before participant recruitment began because this phase started in December 2014. A randomized, double-blind, placebo-controlled crossover trial was designed as a specific trial of an ongoing wider project to investigate the effects of two spinal stimulation currents (TENS vs high frequency stimulation) on neurophysiological reflex measures of excitability. Subjects were allocated to two different therapists (Therapist 1 and Therapist 2, see Fig 1) to compare the intensity applied by each therapist during TENS stimulation. Each participant allocated to each therapist received two interventions (TENS and sham stimulation) so that the effectiveness of TENS on H-reflex modulation could be compared within subjects. TENS and sham stimulation were applied in random order using a web page tool (www.randomizer.org). Therapist 1 and Therapist 2 were two experienced physiotherapists who were instructed to apply both TENS and sham stimulation to their own group of subjects. Both therapists followed the same guidelines for the TENS intervention, with the intensity defined as the stimulus necessary to evoke an innocuous sensation described as “strong but comfortable, just below the motor threshold”. The duration of the intervention was 40min [19, 24, 26–28], and three measurements of Soleus H-reflex amplitude were registered throughout the experimental session: i) before the intervention, ii) during the intervention, 33min after the onset of the stimulus, and iii) 10min following the end of the intervention [19, 26]. The measurement at 33 min after the onset of the stimulus was chosen to ensure an H-reflex recording during the stimulation, respecting a minimum period of 30min after the onset of the stimulus [19, 24, 26], and taking into account that the assessment protocol lasted about 7min. A washout period of 48h was observed between TENS and sham stimulation interventions, longer than the interval time used in other similar previous studies [9, 29–31]. The assessor was the same for all the experiments and was blinded to both interventions but not for the therapist allocation. Participants were blinded by using the same stimulator device, same cables, and same electrode placement and they were not allowed to see the device screen. Participants were also blinded to the hypothesis of the study and they were told that we were testing 2 types of currents which they could feel or not.

### Participants and setting

Twenty healthy volunteers between 18 and 60 years old were recruited through non-probabilistic convenience sampling. The exclusion criteria included participants with musculoskeletal pathology of the lower limbs, history of neuromuscular disease, unable to tolerate electrical current, allergy to the electrode material, pacemaker or any other implanted device, epilepsy, neurotrauma, recent surgical procedures or pain affecting the lower limbs or lower back, diabetes, pregnancy, and cancer. Participants were allocated to Therapist 1 (n = 10) or Therapist 2 (n = 10), were informed of the experimental protocol, and gave written informed consent. All experimental sessions were performed in a clinical laboratory at a stable room temperature in the range of 22–26°C.

### Intervention

Participants were examined in a prone position with their right lower limb knee joint flexed at 120°. Two surface self-adhesive electrodes (9x5cm) (ValuTrode, Axelgaard Manufacturing Co., Ltd, Fallbrook, USA) were fixed on the muscle belly, over the Soleus metamere (S1–S2), corresponding to the vertebral level T10–12 [32].

*TENS stimulation*: Each therapist used the same electrotherapy device (Myomed 932. Enraf Nonius b. V. Vareseweg 127 P.0. Box 12080 NL-3004 GB Rotterdam, Netherlands), applying a constant voltage (CV) and a symmetric biphasic current of 200-μs pulse-width at a frequency of 100 Hz, which was calibrated using a digital oscilloscope before the stimulation session, to their assigned group of participants. The TENS intensity was set to evoke a sensation characterized as “strong but comfortable, just below the motor threshold”. The stimulation intensity was gradually increased until a minimal local muscle contraction was observed and subsequently decreased until the muscle contraction disappeared. The TENS stimulus was adjusted so that the defined perceived sensation was maintained throughout the session. To avoid habituation to the stimulus, participants were asked every 2min [14, 33] to corroborate the perceived sensation, and the intensity was increased if requested.

*Sham stimulation*: Each therapist also applied a sham stimulus intervention. The intensity used for TENS was used for the sham stimulus based on the subject´s sensory perception of the stimulus, which was then decreased to zero where it was fixed until the end of the session. The therapist informed the participant that, in this case, the stimulus intensity could be below the sensory threshold, with the possibility that the participant may or may not feel the current. This method has been validated as an appropriate sham [34, 35] and has been used in previous studies [33].

### Outcome measures

Current density, measured in mA/cm^2^, which reflects the applied current intensity divided by the electrode area (45cm^2^), was recorded by Therapist 1 and Therapist 2 at the onset of the TENS session and at the end, including small readjustments for intensity that were made every 2min in order to maintain the same perceived level of stimulation intensity. The soleus (SOL) H-reflex was elicited with 1ms electrical rectangular pulses (DS7A, Digitimer Ltd., 37 hydeway Welwyn, Garden City Hertfordshire AL7 3 BE, UK) applied to the tibial nerve using a bipolar electrode placed in the popliteal fossa. Electromyography activity (EMG) was recorded with surface bipolar silver chloride electrodes (Signal Conditioning Electrodes v2.3, Delsys Inc., USA) (1000x amplification) and filtered with a built-in 20–450 Hz bandpass filter. EMG electrodes were placed over the Soleus muscle belly at 2/3 of the line between the medial condyle of femur to the medial malleolus following the SENIAM recommendations (http://www.seniam.org/). EMG activity was sampled at 10KHz (MicroPlus 1401, Cambridge Electronic Design, The Science Park, Milton Road, Cambridge CB4 0FE, UK). Maximal peak-to-peak Soleus H-reflex (Hmax) amplitude was registered after increasing tibial nerve stimulation in steps of 1mA. Maximal M wave peak-to-peak amplitude (Mmax) was also annotated by the same procedure. A normalized H-reflex response (Hnor) was also recorded at a test reflex size adjusted to 15% of Mmax because test reflexes recorded at this amplitude have previously been shown to be the most sensitive to facilitatory and inhibitory neuromodulatory input [36].

### Data analysis

The Kolmogorov-Smirnov test was performed to determine the Gaussian distribution of each group of data. A visual inspection of the data distribution and the Q-Q plots was also performed to support these information. The Student’s *t*-test was performed to compare participant characteristics (age, height, weight, and body mass index) and to compare baseline results. The chi-square test was applied to analyze nominal measures such as gender. Due to the evidenced normal distribution of the data (Kolmogorov-Smirnov test *p*>0.05), a two-way repeated-measures ANOVA, with the factor “time” (stimulation onset and termination) and independent factors such as “therapist” (Therapist 1 and Therapist 2) was performed to compare differences in the applied current density. The effect of both TENS and Sham interventions was calculated with the difference between the data of “during” or “post-test” minus the data of “pre-test”. To compare the effect of the TENS intervention on SOL H-reflex amplitude, a two-way repeated-measures ANOVA with the factor “intervention” (TENS and Sham) and independent factor “therapist” (Therapist 1 and Therapist 2) was performed. Bonferroni’s post hoc multiple comparison test was used to reveal specific differences between groups. A Pearson’s correlation test was performed to examine the relationship between applied current density and the effect on SOL H-reflex activity. A *P* value of ≤0.05 was considered to indicate statistical significance. A “posteriori” power analysis was performed to quantify the chance of committing type-II errors. For this, we took into account a bilateral approach, a security level of 95% and a minimum difference to detect of 0.175mV, which correspond to ~20–25% of change.

## Results

### Participant characteristics

All raw data regarding this study are available as supporting information in S1 File. Twenty subjects were allocated to either Therapist 1 (n = 10) or Therapist 2 (n = 10). Both therapists applied TENS and sham stimulation sessions in a randomized order. Finally, one subject assigned to Therapist 2 was eliminated from the study as only one session was completed (Fig 1). The overall selected sample included 11 males and 8 females with a mean age of 28.4 years (SD 11.8), a mean weight of 66.2 kg (SD 11.0), a mean height of 1.70m (SD 0.10), and a mean body mass index of 22.7kg/m^2^ (SD 2.5). Table 1 shows subject-specific physical data grouped by therapist allocation. No significant differences were found between the characteristics of participants assigned to Therapist 1 and Therapist 2.

**Table 1:** Physical characteristics of participants and the comparison between subjects allocated to Therapist 1 and 2.

	Total (n = 19)	Therapist 1 (n = 10)	Therapist 2 (n = 9)	P value
Age *(yr)*, mean (SD)	28.3 (11.8)	27.5 (7.2)	29.3 (15.9)	0.20
Gender, n males (%)	11 (58%)	7 (70%)	4 (40%)	0.29
Weight *(kg)*, mean (SD)	66.2 (10.9)	67.8 (10.9)	64.4 (11.4)	0.52
Height *(m)*, (SD)	1.70 (0.1)	1.72 (0.1)	1.69 (0.12)	0.54
BMI *(kg/m^2^)*, (SD)	22.7 (2.5)	22.8 (2.3)	22.5 (2.8)	0.77

Data are described as mean (SD) for quantitative measures and as number (%) for gender. BMI: Body Mass Index.

### Current density of TENS stimulation

**Fig 2:**
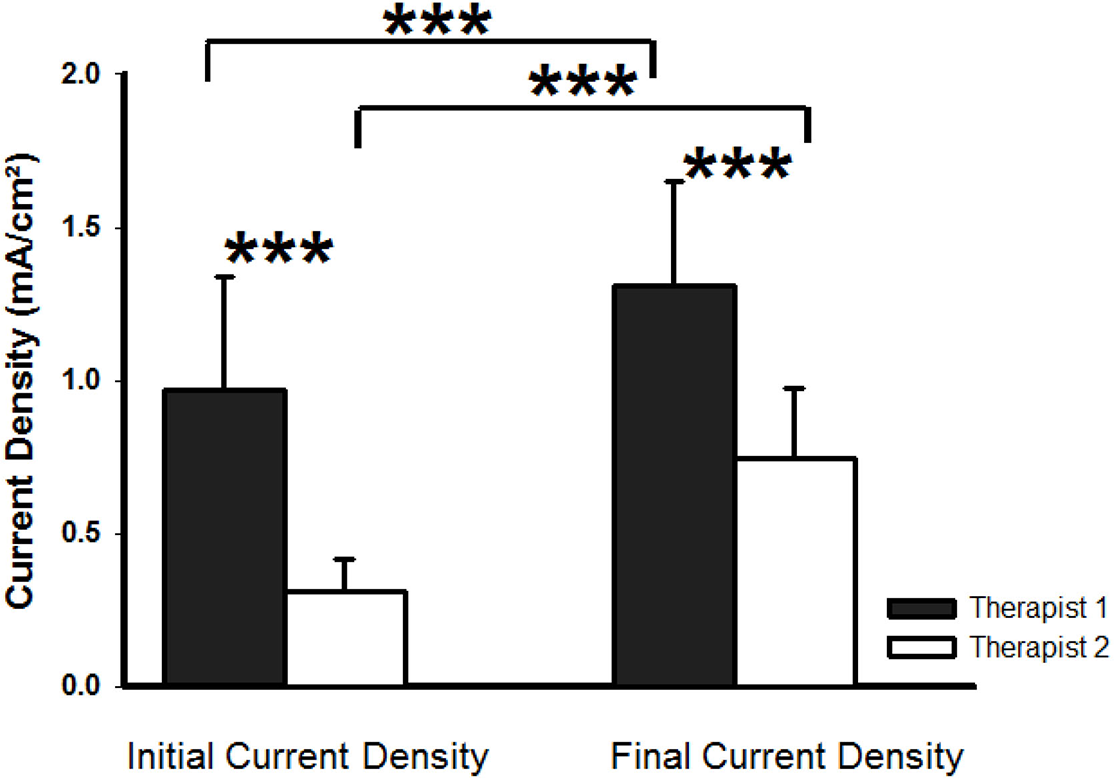
Current density. Current density (mA/cm^2^) applied by Therapist 1 (black bars) and by Therapist 2 (white bars) at the onset and the end of the TENS stimulation session. Data are represented as mean and standard deviation of current density. *** *p*<0.001.

TENS current density showed differences over time (F_(1,18)_ = 68.1; *p*<0.001), which reflected the increase in intensity made during the session by both therapists to avoid habituation (mean increase, 0.41 mA/cm^2^ (SD 0.21); *p*<0.001). The increase in current density made during the TENS intervention by Therapist 1 was 0.34mA/cm^2^ (SD 0.30) (*p*<0.001), and by Therapist 2, it was 0.44mA/cm^2^ (SD 0.30) (*p*<0.001). For the “therapist” factor, significant differences were also observed (F_(1,18)_ = 26.29; *p*<0.001). Therapist 1 applied a higher current density than Therapist 2: at the onset of the stimulation session, Therapist 1 applied a current that was 0.66mA/cm^2^ (SD 0.54) (*p*<0.001) higher than that applied by Therapist 2, and at the end of the stimulation, a difference of 0.56mA/cm^2^ (SD 0.57) (*p*<0.001) was evidenced (Fig 2). No significant differences were observed in the interaction “therapist-time” (F_(1,18)_ = 1,7 p = 0,206).

### Effect of TENS on Soleus H-reflex

Soleus Mmax amplitude remained stable in all trials performed by both therapists. No significant differences were observed in any comparison during the intervention, neither considering the “therapist” factor (F_(1,17)_ = 0.07; *p* = 0.78) neither for the “intervention” factor (F_(1,17)_ = 0.56; *p* = 0.46), nor the interaction “therapist-intervention” (F_(1,17)_ = 0.00 *p* = 0,98). Furthermore, no significant differences were observed in Soleus Mmax 10min after the end of the stimulation session for the “therapist” factor (F_(1,17)_ = 1.33; *p* = 0.26) neither for the “intervention” factor (F_(1,17)_ = 2.92; *p* = 0.10), nor the interaction “therapist-intervention” (F_(1,17)_ = 0.03 *p* = 0,86).

**Fig 3:**
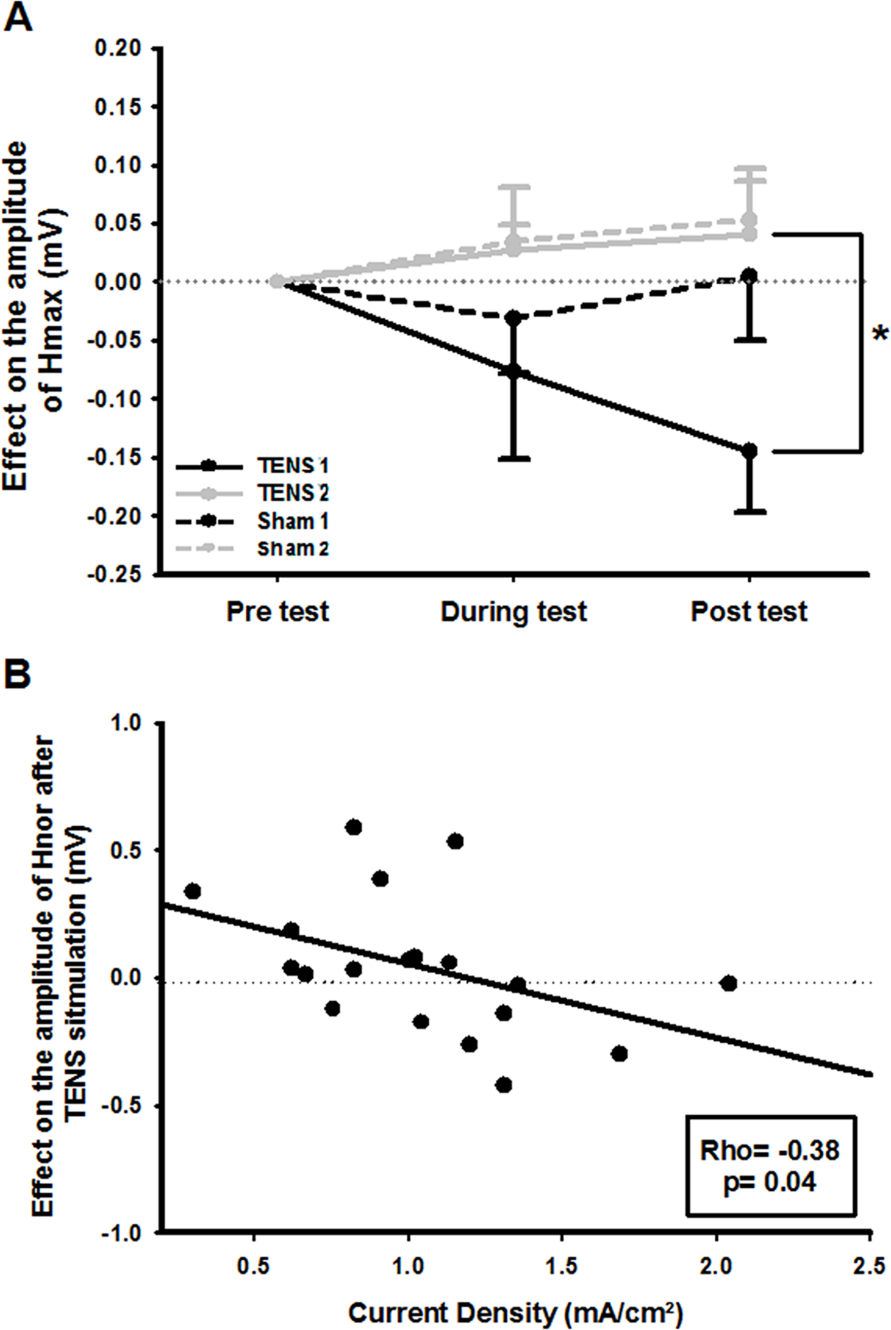
**A)** Effect of TENS and sham stimulation applied by Therapist 1 and Therapist 2 on the amplitude of the Hmax reflex (During/Post-Test minus Pre-Test). *: *p*<0.05. **B)** Correlation between H-reflex and current density. Soleus Hnor reflex amplitude recorded after 10min at the end of the TENS stimulation was characterized by a significant negative correlation with current density applied at the T12 level by both therapists. Positive values indicate excitation and negative values indicate inhibition of the normalized Soleus H-reflex.

Table 2 shows absolute Hmax reflex amplitude following the intervention grouped by therapist and Fig 3A shows the effect of the stimulation from the baseline on the amplitude of the Hmax. Subject #5 from Therapist 2 group was removed for this outcome due to an outlier data. No significant differences were observed in Hmax at the baseline between TENS applied by Therapist 1 and TENS applied by Therapist 2 (*p* = 0.35), between TENS applied by Therapist 1 and sham applied by Therapist 1 (*p* = 0.74), and between TENS applied by Therapist 2 and sham applied by Therapist 2 (*p* = 0.68). No significant difference was observed during stimulation considering the “therapist” factor (F_(1,16)_ = 2.58, *p* = 0.1) neither for the “intervention” factor (F_(1,16)_ = 0.23; *p* = 0.64) nor the interaction “therapist-intervention” (F_(1,16)_ = 0.12; *p* = 0.74) and power analysis revealed a statistical power between 53% (TENS 1 vs sham 1 comparison) and 98% (TENS 2 vs sham 2 comparison). However, when measured 10min after stimulation, a significant difference for the effect of the “therapist” factor on Hmax was observed (F_(1,16)_ = 9.11; *p*<0.008), but nor for “intervention” factor (F_(1,16)_ = 1.78; *p* = 0.2) neither nor the interaction “therapist-intervention” (F_(1,16)_ = 1.27; *p* = 0.28). For TENS, comparison between therapists showed that Therapist 1, who applied a higher current density, produced greater inhibition of the Hmax amplitude (0.19mV, SD 0.31; *p* = 0.03; power = 69%) than Therapist 2. For sham stimulation, no significant difference was observed between therapists. When the effect of TENS was compared with that of sham, Therapist 1 revealed a trend for inhibition of Hmax (0.15mV, SD 0.34; *p* = 0.08; power = 65%) while no difference was observed for Therapist 2 (0.01mV, SD 0.37; *p* = 0.89; power = 87%).

**Table 2:** Peak-to-peak Soleus H reflex amplitude (Hmax), following TENS or sham stimulation and comparison between interventions.

	Interventions (mV) Mean (SD)			Difference within Intervention (mV) Mean (95% CI)			Comparison between Interventions (mV) Mean (SD)	
	Pre-test	During- test	Post- test	During-test minus Pre-test	Post-test minus Pre-test		During-test effect	Post-test effect
**TENS 1**	0.70 (0.45)	0.62 (0.38)	0.55 (0.33)	-0.08 (-0.2 to 0.05)	-0.15 (-0.2 to 0.04)	TENS 1 vs TENS 2	-0.11 (0.35) p = 0.25	-0.19 (0.31) p = 0.03*
**TENS 2**	0.52 (0.29)	0.55 (0.31)	0.56 (0.31)	0.03 (-0.1 to 0.2)	0.04 (-0.08 to 0.2)			
**SHAM 1**	0.67 (0.40)	0.64 (0.39)	0.67 (0.42)	-0.03 (-0.1 to 0.06)	0.00 (-0.09 to 0.1)	TENS 1 vs SHAM 1	-0.05 (0.31) p = 0.55	-0.15 (0.34) **p = 0.08**
**SHAM 2**	0.59 (0.63)	0.62 (0.52)	0.64 (0.60)	0.03 (-0.08 to 0.2)	0.05 (-0.05 to 0.2)	TENS 2 vs SHAM 2	0.00 (0.35) p = 0.93	-0.01 (0.37) p = 0.89

TENS 1 = TENS applied by Therapist 1; TENS 2 = TENS applied by Therapist 2; SHAM 1 = sham stimulation applied by Therapist 1; SHAM 2 = sham stimulation applied by Therapist 2.

*: p<0.05; p≤0.08 are shown in bold.

Table 3 shows values of Hnor amplitude following the intervention. No significant differences were observed in Hnor at the baseline between TENS applied by Therapist 1 and TENS applied by Therapist 2 (*p* = 0.47), between TENS applied by Therapist 1 and sham applied by Therapist 1 (*p* = 0.72), and between TENS applied by Therapist 2 and sham applied by Therapist 2 (*p* = 0.54). No difference for Hnor amplitude during stimulation was found for the “therapist” factor (F_(1,17)_ = 3.35; *p* = 0.08) neither nor for the “intervention” factor (F_(1,17)_ = 1.02; *p* = 0.33), nor the interaction “therapist-intervention” (F_(1,17)_ = 0.02; *p* = 0.88) and power analysis revealed a statistical power between 31% (TENS 2 vs sham 2 comparison) and 56% (TENS 1 vs TENS 2 comparison). However, Hnor amplitude measured at 10min after the end of the stimulation showed differences for the “therapist” factor (F_(1,17)_ = 4.59; *p* = 0.047), but not for “intervention” factor (F_(1,17)_ = 3.06; *p* = 0.09) neither nor the interaction “therapist-intervention” (F_(1,17)_ = 0.66; *p* = 0.43). When TENS was compared across therapists, Therapist 1 revealed a trend for greater reflex inhibition (0.24mV, SD 0.48; *p* = 0.06; power = 31%) compared with Therapist 2. Following sham stimulation, no differences between therapists were identified for Hnor amplitude. When the effect of TENS and sham were compared, a trend for Hnor reflex inhibition was identified for Therapist 1 (0.15mV, SD 0.36; *p* = 0.08; power = 43%).

**Table 3:** Peak-to-peak Hnor amplitude following TENS or sham stimulation and comparison between interventions.

	Interventions (mV) Mean (SD)			Difference within Intervention (mV). Mean (95% CI)			Comparison between Interventions (mV). Mean (SD)	
	Pre-test	During-test	Post- test	During-test minus Pre-test	Post-test minus Pre-test		During-test effect	Post-test effect
**TENS 1**	0.40 (0.26)	0.47 (0.35)	0.33 (0.26)	0.07 (-0.1 to 0.2)	-0.07 (-0.2 to 0.1)	TENS 1 vs TENS 2	-0.09 (0.50) p = 0.70	-0.24 (0.48) **p = 0.06**
**TENS 2**	0.33 (0.10)	0.49 (0.28)	0.50 (0.29)	0.16 (-0.01 to 0.3)	0.17 (0.0 to 0.3)			
**SHAM 1**	0.42 (0.26)	0.53 (0.33)	0.50 (0.34)	0.11 (-0.05 to 0.3)	0.08 (-0.04 to 0.2)	TENS 1 vs SHAM 1	-0.04 (0.44) p = 0.65	-0.15 (0.36) **p = 0.08**
**SHAM 2**	0.30 (0.18)	0.56 (0.49)	0.53 (0.39)	0.26 (0.1 to 0.4)	0.23 (0.1 to 0.4)	TENS 2 vs SHAM 2	-0.10 (0.46) p = 0.41	-0.06 (0.38) p = 0.53

TENS 1 = TENS applied by Therapist 1; TENS 2 = TENS applied by Therapist 2; SHAM 1 = sham stimulation applied by Therapist 1; SHAM 2 = sham stimulation applied by Therapist 2. p≤0.08 are shown in bold

Correlational analysis between current density applied by both therapists and the effect on normalized SOL H-reflex amplitude revealed a significant relationship 10min after TENS with reflex inhibition (Rho = -0.38; *p* = 0.04, see Fig 3B). However, no significant correlations were found regarding the effect on Hmax during stimulation nor after 10min of TENS.

## Discussion

The present study shows a significant difference in TENS current density applied by two independent therapists who received the same instructions to adjust the intensity level to evoke a “strong but comfortable sensation, just below motor threshold”. In addition, the subject group that received the higher TENS current density revealed a greater inhibition of SOL H-reflex amplitude when compared with the application of the sham stimulation and with those participants who received TENS with a lower current density. These findings support the need for a change in the paradigm of standardizing TENS intensity, which is currently based on subjective assessment of the evoked sensation, to one that requires objective calculation of stimulation based on current density, which in turn will help to demonstrate the effectiveness of this intervention for the neuromodulation of different pathologies. The adjustment of TENS intensity is usually based on subjective verbal commands in both clinical practice and scientific studies [9, 11, 12, 37]. Very few authors indicate absolute values of the intensity of the current applied and furthermore, do not provide information regarding the size of the electrodes [15, 20], which makes it impossible to standardize the amount of current. The importance of quantifying TENS intensity and normalizing it to the size of the electrodes by calculating current density has been raised previously[33]. A previous study in transcranial direct current stimulation showed more discomfort with larger electrodes using the same current density [38], which may reflect enhanced spatial summation by an increased area of stimulated nerve endings. More studies in the field of TENS are needed to understand the specific role of electrode size to produce better neuromodulatory effects without discomfort.

Some studies have emphasized the importance of standardizing current intensity for TENS, which shows a direct dose-dependent effect in models of experimental pain, where higher intensity levels produce greater effects [5, 8–12]. Our study shows how TENS is only effective in reducing SOL H-reflex amplitude when greater current densities (Therapist 1: 1.30, SD 0.34mA/cm^2^) are applied, even though both therapists assumed that they were applying the same current intensity. Different effects of TENS depending on the applied current intensity has been shown in numerous clinical situations. A systematic review of TENS for osteoarthritic knee pain concluded that TENS was more effective for pain relief at higher intensities [39], supporting the results obtained in this study. In contrast, a similar systematic review assessing TENS for the same pathology, and which failed to account for the current intensity, showed inconclusive results [40]. In specific studies where TENS intensity was registered objectively, current densities applied at similar values to those in the present study (1.77mA/cm^2^ [33] and 1.56mA/cm^2^ [5]) demonstrated favorable results for the treatment of experimental pain outcome measures. Habituation is a common phenomenon associated with stimulation procedures that need periodic readjustment of the current intensity to achieve the same level of evoked sensation to the stimulus. This stimulus readjustment has been shown to be more effective than maintaining the applied stimulus at a constant intensity [12]. The paucity of studies identifying significant neuromodulatory effects of TENS on H-reflex amplitude may reflect the failure to readjust current intensity during the session [15, 16, 19–21, 24, 41]. Furthermore, in cases where normal sensory function is altered or lost following pathology, standardizing TENS current intensity is an additional challenge [15]. In the present study, the protocol employed by Claydon et al. [14], in which current intensity was adjusted every 2min, was used. Current density applied by both therapists significantly increased over the 40min session, suggesting that habituation was present during TENS stimulation. The role of habituation and the necessity of readjusting current intensity throughout the TENS session is an important issue that should be addressed in future studies to determine its effect on H-reflex activity and spinal excitability.

Very few studies have applied TENS at the spinal level. Hofstoetter et al. [26] showed that transcutaneous spinal stimulation clinically improved spasticity but without a concomitant change in SOL H-reflex amplitude, while Simorgh et al. [25] demonstrated that reflex latency increased and SOL H-reflex amplitude decreased following application of a tripolar TENS technique in healthy subjects. The diverse effects on SOL H-reflex activity is mirrored when TENS is applied to the peripheral nervous system. While some studies showed no change in SOL H-reflex [20, 22, 23], others showed a decrease or a tendency for the SOL H-reflex amplitude to decrease [15, 19, 24]. In these TENS studies [15, 19, 20, 22–24], current density was either not calculated according to the size of the electrodes used [15, 19, 20, 24], or the TENS intensities were not reported [22, 23]. The subjective grading of TENS intensity based on the evoked sensation of a “strong but comfortable” sensation [22, 23] or “sensory threshold” were the most prevalent criteria used [19, 24]. Our study only showed a tendency for TENS to modulate H-reflex activity compared with sham stimulation for Therapist 1 (*p* = 0.08). However, a slight low statistical power of 65% could mask a real significant difference. Therefore, it is not possible to draw conclusions regarding the efficacy of TENS for H-reflex modulation. Better standardization of TENS current intensity, without relying on the subjective description of the evoked stimulus should help to more clearly demonstrate its neuromodulatory effects. Importantly, our study showed that higher current densities for both therapists correlated with greater inhibition of Soleus H-reflex activity after TENS.

### Study limitations

This is the first study to address directly the differences between therapists when instructed to apply TENS at an intensity that is corroborated subjectively by the subject. However, several methodological issues should be improved in future studies. Firstly, although the order of the interventions applied by each therapist was randomized, the therapist allocation was performed in a non-randomized, consecutive order. Although no significant differences were found for subject characteristics when compared between both therapists (see Table 1), the proportion of males/females was not balanced between groups. Furthermore, the range of age in the inclusion criteria (18–60 years) were too high. These factors could affect the TENS perception and the comparison between different groups of people because of bias, but no studies have been found showing differences in the TENS current perception in terms of age or sex. Matched recruitment in terms of sex and age, or a crossover design comparing the same group of people is suggested in future studies.

Another limitation is that the assessor was not blinded to the therapist allocation, although he was blinded to the application of each intervention (TENS or sham). This was performed because “a priori”, both therapists would apply the same intensity based on the same description of a “strong but comfortable” intensity. Regarding to the blinding of the subjects, although this blinding method was widely used before [33, 35] the success of the blinding was not formally tested. Finally, although sample size was not calculated initially, similar studies, which have applied TENS and recorded H-reflex amplitude as the main outcome measure, also recruited a minimal group size of 10 healthy volunteers per group [19, 24, 25]. The statistical power analysis revealed a slightly low power (65%) for the non-differences detected in the comparison between TENS 1 and TENS 2 (p = 0.08) but a high power (89%) for the absence of differences in the comparison between TENS applied by Therapist 2 and sham applied by Therapist 2 ten minutes after the stimulation on the Hmax outcome.

## Conclusions

Application of TENS intensity based on the subjective perception of the stimulus made by the participant was differently interpreted by the two therapists. Furthermore, the effectiveness of TENS stimulation on SOL H-reflex amplitude modulation depended on the stimulus intensity applied with the same pulse-width and frequency. Reliable evaluation of TENS intensity should be based on current density calculation and should be routinely documented for TENS sessions to permit comparisons among different studies and to determine the real effectiveness of TENS. Further studies are required to determine the relationship between TENS intensity and differential spinal excitability modulation using accurately determined TENS current density.

## Supporting information

S1 File Raw data of all subjects recruited in the study. Each label contains the whole information regarding to: subjects characteristics; density current outcome; Mmax outcome; Hmax outcome; Hnor outcome; and the power analysis performed.
(XLSX)
